# Sphingosine-1-Phosphate Mediates ICAM-1-Dependent Monocyte Adhesion through p38 MAPK and p42/p44 MAPK-Dependent Akt Activation

**DOI:** 10.1371/journal.pone.0118473

**Published:** 2015-03-03

**Authors:** Chih-Chung Lin, I-Ta Lee, Chun-Hao Hsu, Chih-Kai Hsu, Pei-Ling Chi, Li-Der Hsiao, Chuen-Mao Yang

**Affiliations:** 1 Department of Anesthetics, Chang Gung Memorial Hospital at Lin-Kou and College of Medicine, Chang Gung University, Kwei-San, Tao-Yuan, Taiwan; 2 Department of Physiology and Pharmacology and Health Ageing Research Center, College of Medicine, Chang Gung University, Kwei-San, Tao-Yuan, Taiwan; Faculty of Medicine & Health Sciences, UNITED ARAB EMIRATES

## Abstract

Up-regulation of intercellular adhesion molecule-1 (ICAM-1) is frequently implicated in lung inflammation. Sphingosine-1-phosphate (S1P) has been shown to play a key role in inflammation via adhesion molecules induction, and then causes lung injury. However, the mechanisms underlying S1P-induced ICAM-1 expression in human pulmonary alveolar epithelial cells (HPAEpiCs) remain unclear. The effect of S1P on ICAM-1 expression was determined by Western blot and real-time PCR. The involvement of signaling pathways in these responses was investigated by using the selective pharmacological inhibitors and transfection with siRNAs. S1P markedly induced ICAM-1 expression and monocyte adhesion which were attenuated by pretreatment with the inhibitor of S1PR1 (W123), S1PR3 (CAY10444), c-Src (PP1), EGFR (AG1478), PDGFR (AG1296), MEK1/2 (U0126), p38 MAPK (SB202190), JNK1/2 (SP600125), PI3K (LY294002), or AP-1 (Tanshinone IIA) and transfection with siRNA of S1PR1, S1PR3, c-Src, EGFR, PDGFR, p38, p42, JNK1, c-Jun, or c-Fos. We observed that S1P-stimulated p42/p44 MAPK and p38 MAPK activation was mediated via a c-Src/EGFR and PDGFR-dependent pathway. S1P caused the c-Src/EGFR/PDGFR complex formation. On the other hand, we demonstrated that S1P induced p42/p44 MAPK and p38 MAPK-dependent Akt activation. In addition, S1P-stimulated JNK1/2 phosphorylation was attenuated by SP600125 or PP1. Finally, S1P enhanced c-Fos mRNA levels and c-Jun phosphorylation. S1P-induced c-Jun activation was reduced by PP1, AG1478, AG1296, U0126, SP600125, SB202190, or LY294002. These results demonstrated that S1P-induced ICAM-1 expression and monocyte adhesion were mediated through S1PR1/3/c-Src/EGFR, PDGFR/p38 MAPK, p42/p44 MAPK/Akt-dependent AP-1 activation.

## Introduction

Lung inflammation is a pivotal event in the pathogenesis of chronic obstructive pulmonary disease and asthma. These inflammatory responses are mediated by complex interactions between both circulating polymorphonuclear cells (PMNs) and the vascular endothelium. Several studies indicate that expression of adhesion molecules on the cell surface of endothelial cells plays a critical role in the inflammatory responses [1]. Raised levels of adhesion molecules might contribute to the recruitment of PMNs to the regions of inflammatory tissue. These adhesion molecules are classified into two major families: the Ig superfamily (e.g., ICAM-1 and VCAM-1) and the selectins (e.g., P-selectin and E-selectin) [2]. ICAM-1 is an inducible cell surface glycoprotein on several cell types, which mediates the tight adhesiveness of PMNs and thus facilitates PMNs migration across the vascular endothelium barrier and then interacts with lung epithelium [3].

Sphingosine 1-phosphate (S1P) is a bioactive sphingolipid metabolite that plays important roles in allergic responses, including asthma and anaphylaxis [4]. S1P regulates numerous cellular responses, including motility and cytoskeletal rearrangements, formation of adherens junctions, proliferation, survival, angiogenesis, and the trafficking of immune cells [5]. These myriad effects are partly elicited by binding of S1P to a family of five G protein-coupled receptors (S1PRs), termed S1PR1–5. Moreover, S1P has been shown to induce lung injury and inflammation [6]. In addition, S1P has been also shown to induce ICAM-1 or VCAM-1 expression in various cell types [7,8]. However, the mechanisms of S1P-regulated ICAM-1 expression in human pulmonary alveolar epithelial cells (HPAEpiCs) are not completely understood. Thus, to clarify the mechanisms of ICAM-1 induction by S1P in lung epithelium was recognized as a new therapeutic approach in the management of respiratory diseases.

c-Src, a common modular participating in the crosstalk between the cytoplasmic protein tyrosine kinases and receptors, has been shown to mediate ICAM-1 expression in various cell types [9,10]. On the other hand, previous studies indicated that c-Src regulates platelet-derived growth factor receptor (PDGFR) and epidermal growth factor receptor (EGFR) transactivation [11], which further promotes inflammatory responses. Mitogen-activated protein kinases (MAPKs) are important components of signaling modules activated by neurotransmitters, cytokines, and growth factors, as well as chemical and mechanical stressors. MAPKs are also implicated in S1P-induced inflammatory responses [12,13]. Recent studies suggested that numerous components of the PI3K/Akt pathway play a crucial role in the expression and activation of inflammatory mediators, inflammatory cell recruitment, immune cell function, airway remodeling, and corticosteroid insensitivity in chronic inflammatory respiratory diseases [2]. Indeed, previous studies indicated that PI3K/Akt regulates the expression of adhesion molecules in various cell types [10,14]. S1P has been shown to enhance Akt activation [15,16]. Although these studies have demonstrated that ICAM-1 induction was regulated via various signaling components, whether these signalings also participated in ICAM-1 expression and monocyte adhesion on HPAEpiCs challenged with S1P remains unknown.

The ICAM-1 promoter has been shown to contain several binding sequences for various transcription factors, including AP-1 [17]. AP-1 is a heterogeneous collection of dimeric transcription factors comprising Jun, Fos, and ATF subunits. Among AP-1 subunits, c-Jun is the most important transcriptional activator in inflammatory status [2]. AP-1 activity is regulated by multiple mechanisms, including phosphorylation by various MAPKs [18]. Thus, in this study, we also investigated the role of AP-1 in ICAM-1 expression in HPAEpiCs challenged with S1P.

In addressing these questions, experiments were undertaken to investigate the effects of S1P on expression of ICAM-1 and monocyte adhesion on HPAEpiCs. These findings suggest that the increased expression of ICAM-1 and monocyte adhesion on S1P-challenged HPAEpiCs are mediated through S1PR1/3/c-Src/EGFR, PDGFR/p38 MAPK, p42/p44 MAPK/Akt-dependent AP-1 activation. These results provide new insights into the mechanisms of S1P action on HPAEpiCs to regulate the expression of ICAM-1 and thus exaggerate the inflammation responses.

## Materials and Methods

### Materials

Anti-ICAM-1, anti-GAPDH, anti-S1PR1, anti-S1PR2, anti-S1PR3, anti-c-Src, anti-EGFR, anti-PDGFR, anti-JNK1, anti-p42, anti-p38, anti-c-Jun, and anti-c-Fos antibodies and ICAM-1 neutralizing antibody were from Santa Cruz Biotechnology (Santa Cruz, CA). Anti-phospho-c-Src, anti-phospho-EGFR, anti-phospho-PDGFR, anti-phospho-JNK1/2, anti-phospho-p42/p44 MAPK, anti-phospho-p38 MAPK, anti-phospho-Akt, and anti-phospho-c-Jun antibodies were from Cell Signaling (Danver, MA). W123, JTE-013 and CAY10444 were from Cayman (Ann Arbor, MI). PP1, U0126, SP600125, SB202190, AG1478, AG1296, Genistein, Tanshinone IIA, and LY294002 were from Biomol (Plymouth Meetings, PA). BCECF/AM was from Molecular Probes (Eugene, OR). SDS-PAGE reagents were from MDBio Inc (Taipei, Taiwan). All other reagents were from Sigma (St. Louis, MO).

### Cell culture

Human pulmonary alveolar epithelial cells (HPAEpiCs) were purchased from the ScienCell Research Laboratories (San Diego, CA) and grown as previously described [19]. Experiments were performed with cells from passages 4 to 8. Treatment of HPAEpiCs with DMSO or the pharmacological inhibitors alone had no significant effect on cell viability determined by a 2,3-bis-(2-methoxy-4-nitro-5-sulfophenyl)-2H-tetrazolium-5-carboxanilide (XTT) assay (data not shown).

### Transient transfection with siRNAs

Human siRNAs of scrambled, S1PR1, S1PR3, c-Src, EGFR, PDGFR, p38, p42, JNK1, c-Jun, and c-Fos were from Sigma (St. Louis, MO). Transient transfection of siRNAs was carried out using Metafectene transfection reagent (Biontex, Germany). siRNA (100 nM) was formulated with Metafectene transfection reagent according to the manufacturer’s instruction.

### Western blot

Growth-arrested cells were incubated with S1P at 37°C for the indicated time intervals. The cells were washed, scraped, collected, and centrifuged at 45000 × *g* at 4°C for 1 h to yield the whole cell extract, as previously described [19]. Samples were denatured, subjected to SDS-PAGE using a 10% running gel, and transferred to nitrocellulose membrane. Membranes were incubated with an anti-ICAM-1 antibody for 24 h, and then membranes were incubated with an anti-rabbit horseradish peroxidase antibody for 1 h. The immunoreactive bands were detected by ECL reagents.

### Real-time PCR

Total RNA was extracted using TRIzol reagent. mRNA was reverse-transcribed into cDNA and analyzed by real-time PCR using SYBR Green PCR reagents (Applied Biosystems, Branchburg, NJ) with primers specific for ICAM-1, c-Fos, c-Jun, and GAPDH. The levels of ICAM-1, c-Fos, and c-Jun expression were determined by normalizing to that of GAPDH expression.

### RT-PCR analysis

Total RNA was isolated using TRIzol according to the protocol of the manufacturer. The cDNA obtained from 0.5 μg total RNA was used as a template for PCR amplification as previously described [11]. The primers used were as follows:

ICAM-1:

5′-CAAGGGGAGGTCACCCGCGAGGTG-3′ (sense)

5′-TGCAGTGCCCATTATGACTG-3′ (anti-sense)

β-actin:

5′-CTAGAAGCATTTGCGGTGGACGATGGAGGG-3′ (sense)

5′-TGACGGGGTCACCCACACTGTGCCCATCTA-3′ (anti-sense)

S1PR1:

5′-CCACAACGGGAGCAATAACT-3′ (sense)

5′-GTAAATGATGGGGTTGGTGC-3′ (anti-sense)

S1PR2:

5′-GCAGCTTGTACTCGGAGTACCTGAAC-3′ (sense)

5′-CGATGGCCAACAGGATGATGGAGAAG-3′ (anti-sense)

S1PR3:

5′-CGTCTGTGAATGCCAAGTGATGGCAACTG-3′ (sense)

5′-CGAGTTGTTGTGGTTGGCCACCTTACG-3′ (anti-sense)

S1PR4:

5′-ATCACGCTGAGTGACCTGCTCA-3′ (sense)

5′-TGCGGAAGGAGTAGATGA-3′ (anti-sense)

S1PR5:

5′-CTACTGTCGGGGCCGCTCAC-3′ (sense)

5′-CGGTTGGTGAACGTGTAGATGA-3′ (anti-sense)

### Adhesion assay

HPAEpiCs were grown to confluence in 6-well plates, incubated with S1P for 16 h, and then adhesion assays were performed. Briefly, THP-1 cells (human acute monocytic leukemia cell line) were labeled with a fluorescent dye, 10 μM BCECF/AM, at 37°C for 1 h in RPMI-1640 medium (Gibco BRL, Grand Island, NY) and subsequently washed by centrifugation. Confluent HPAEpiCs in 6-well plates were incubated with THP-1 cells (2 × 10^6^ cells/ml) at 37°C for 1 h. Non-adherent THP-1 cells were removed and plates were gently washed twice with PBS. The numbers of adherent THP-1 cells were determined by counting four fields per 200X high-power field well using a fluorescence microscope (Zeiss, Axiovert 200M). Experiments were performed in triplicate and repeated at least three times.

### Co-immunoprecipitation assay

Cell lysates containing 1 mg of protein were incubated with 2 μg of an anti-c-Src antibody at 4°C for 24 h, and then 10 μl of 50% protein A-agarose beads was added and mixed at 4°C for 24 h. The immunoprecipitates were collected and washed thrice with a lysis buffer without Triton X-100. 5X Laemmli buffer was added and subjected to electrophoresis on SDS-PAGE, and then blotted using an anti-EGFR, anti-PDGFR, or anti-c-Src antibody.

### Luciferase activity assay

The human ICAM-1 (pIC-339) firefly luciferase was kindly provided by Dr. P. T. van der Saag (Hubrecht Laboratory, Utrecht, The Netherlands). All plasmids were prepared by using QIAGEN plasmid DNA preparation kits. ICAM-1-luc activity was determined as previously described using a luciferase assay system (Promega, Madison, WI) [3].

#### Analysis of data

All the data were expressed as the mean or mean±S.E.M. The data were analyzed using a GraphPad Prism Program (GraphPad, San Diego, CA) by one-way analysis of variance (ANOVA) followed with Tukey’s post-hoc test. A *P*<0.05 value was considered significant.

## Results

### S1P induces ICAM-1-dependent monocyte adhesion

**Fig 1:**
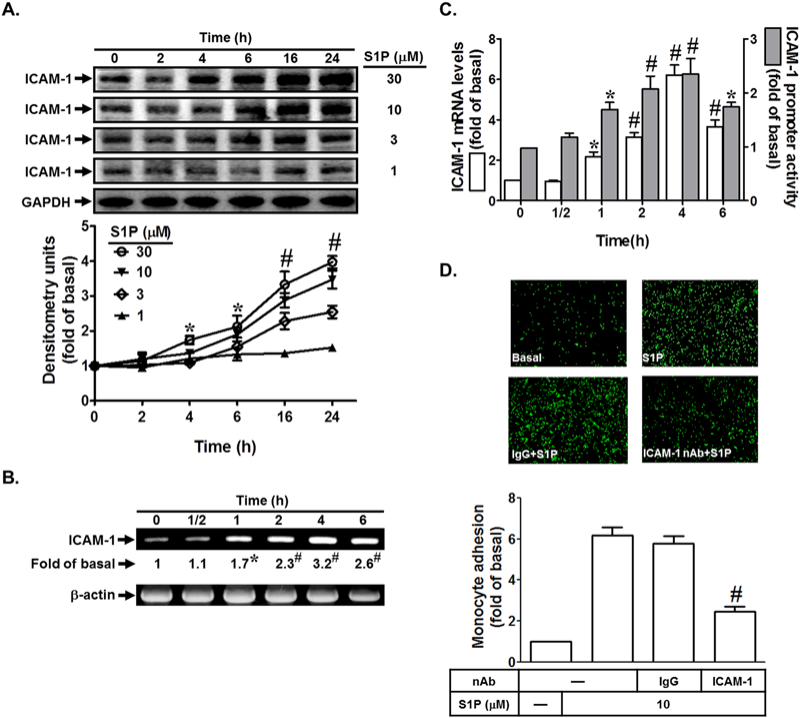
S1P induces ICAM-1-dependent monocyte adhesion. (A) HPAEpiCs were incubated with various concentrations of S1P for the indicated time intervals. ICAM-1 protein expression was detected by Western blot. (B) Cells were incubated with 10 M S1P for the indicated time intervals. The ICAM-1 mRNA expression was determined by RT-PCR. (C) Cells were incubated with 10 M S1P for the indicated time intervals. The ICAM-1 mRNA expression and promoter activity were analyzed by real-time PCR and promoter assay, respectively. (D) Cells were pretreated with an ICAM-1 neutralizing antibody or IgG antibody, and then incubated with S1P for 16 h. Then THP-1 cells adherence was measured. Data are expressed as mean±S.E.M. of three independent experiments. *P<0.05; ^#^ *P*<0.01, as compared with the cells exposed to vehicle alone (A-C) or S1P alone (D).

To investigate the effect of S1P on ICAM-1 expression, HPAEpiCs were incubated with various concentrations of S1P for the indicated time intervals. As shown in Fig. 1A, S1P induced ICAM-1 expression in a time- and concentration-dependent manner. In addition, S1P also enhanced ICAM-1 mRNA expression and promoter activity in a time-dependent manner in HPAEpiCs (Fig. 1B and C). Finally, we demonstrated that adhesion of THP-1 to HPAEpiCs challenged with S1P was enhanced, which was inhibited by an ICAM-1 neutralizing antibody but not by anti-IgG Ab (Fig. 1D). Taken together, we suggest that S1P induces monocyte adhesion via an ICAM-1-dependent pathway in HPAEpiCs.

### S1PR1 and S1PR3 play key roles in S1P-induced ICAM-1 expression

**Fig 2:**
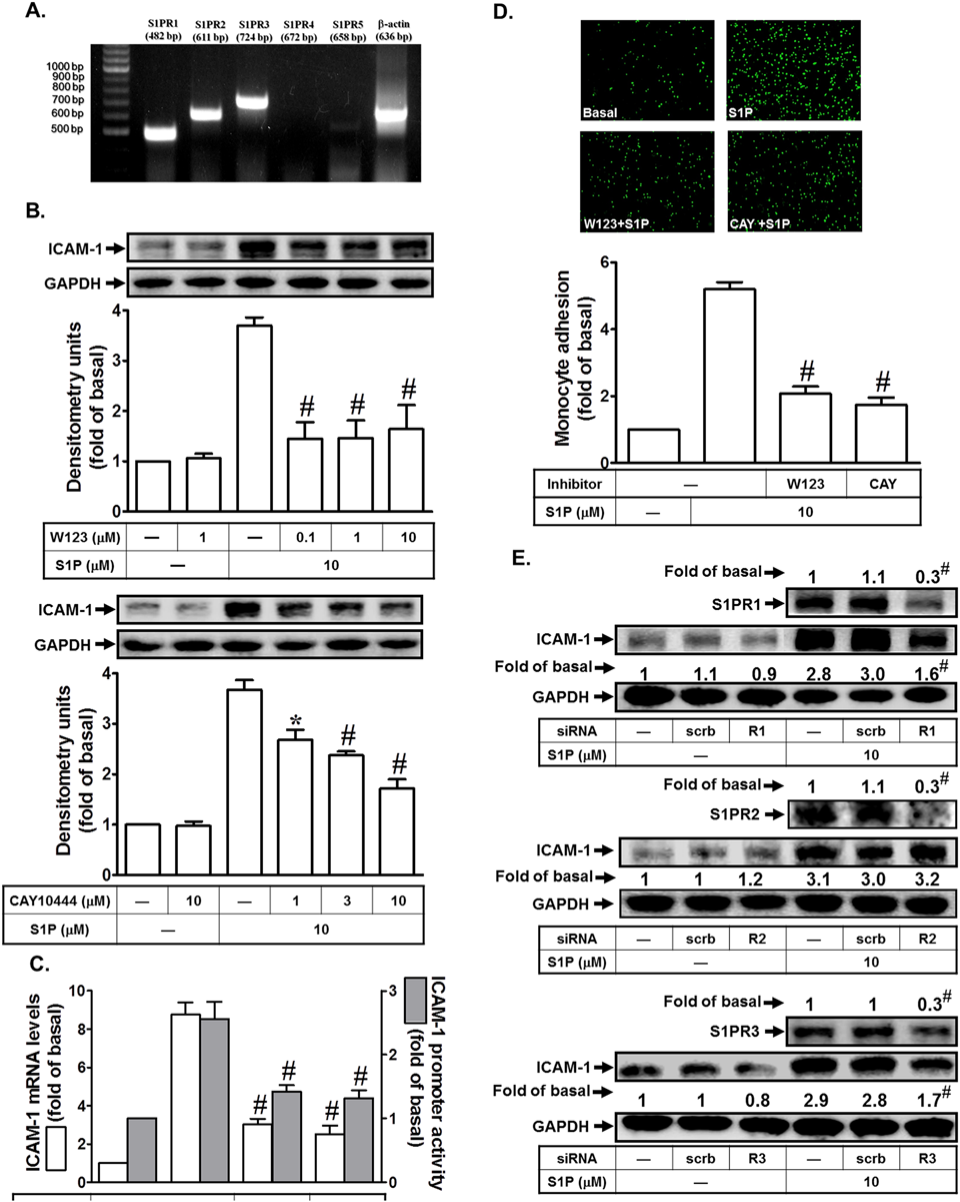
S1P up-regulates ICAM-1 expression via S1PR1 and S1PR3 in HPAEpiCs. (A) The mRNA expression of various S1P receptors on HPAEpiCs was determined by RT-PCR. (B) Cells were pretreated with W123 or CAY10444 for 1 h, and then incubated with S1P for 16 h. The ICAM-1 protein expression was determined by Western blot. (C) Cells were pretreated with W123 (10 M) or CAY10444 (10 M) for 1 h, and then incubated with S1P for 4 h. The ICAM-1 mRNA expression and promoter activity were determined by real-time PCR and promoter assay, respectively. (D) Cells were pretreated with W123 (10 M) or CAY10444 (10 M) for 1 h, and then incubated with S1P for 16 h. The THP-1 cells adherence was measured. (E) Cells were transfected with siRNA of scrambled, S1PR1, S1PR2, or S1PR3, and then incubated with S1P (10 μM) for 16 h. The levels of S1PR1, S1PR2, S1PR3, and ICAM-1 proteins were determined by Western blot. Data are expressed as mean (E) or mean±S.E.M. (B, C, and D) of three independent experiments. *P<0.05; ^#^ *P*<0.01, as compared with the cells exposed to S1P alone (B, C, and D) or transfected with siRNA of scrambled+S1P (E).

S1PR1, S1PR2, and S1PR3 are ubiquitously expressed, whereas the levels of S1PR4 and S1PR5 expression are predominantly existed in immune cells, CNS, and some organs [5]. Which types of S1P receptors expressed on HPAEpiCs are still unclear. Therefore, we identified the expression of S1P receptors on HPAEpiCs. As shown in Fig. 2A, S1PR1, S1PR2, and S1PR3 are expressed on HPAEpiCs, determined by RT-PCR. On the other hand, we observed that pretreatment with the selective inhibitor of S1PR1 (W123) or S1PR3 (CAY10444) markedly reduced S1P-induced ICAM-1 protein levels (Fig. 2B). Indeed, pretreatment with the inhibitor of S1PR2 (JTE-013) had no effect on S1P-induced ICAM-1 expression (data not shown). In addition, pretreatment with W123 or CAY10444 markedly inhibited S1P-induced ICAM-1 mRNA levels and promoter activity (Fig. 2C). These two inhibitors also attenuated the monocyte adhesion to HPAEpiCs challenged with S1P (Fig. 2D). To further confirm the roles of S1PR1 and S1PR3 in S1P-induced ICAM-1 expression, siRNA of S1PR1, S1PR2, or S1PR3 was used. As shown in Fig. 2E, transfection with S1PR1 and S1PR3 but not S1PR2 siRNA significantly reduced S1P-induced ICAM-1 expression in HPAEpiCs. Thus, we demonstrate that S1PR1 and S1PR3 play critical roles in S1P-induced ICAM-1 expression in HPAEpiCs and monocyte adhesion.

### c-Src plays a critical role in S1P-induced ICAM-1 expression

**Fig 3:**
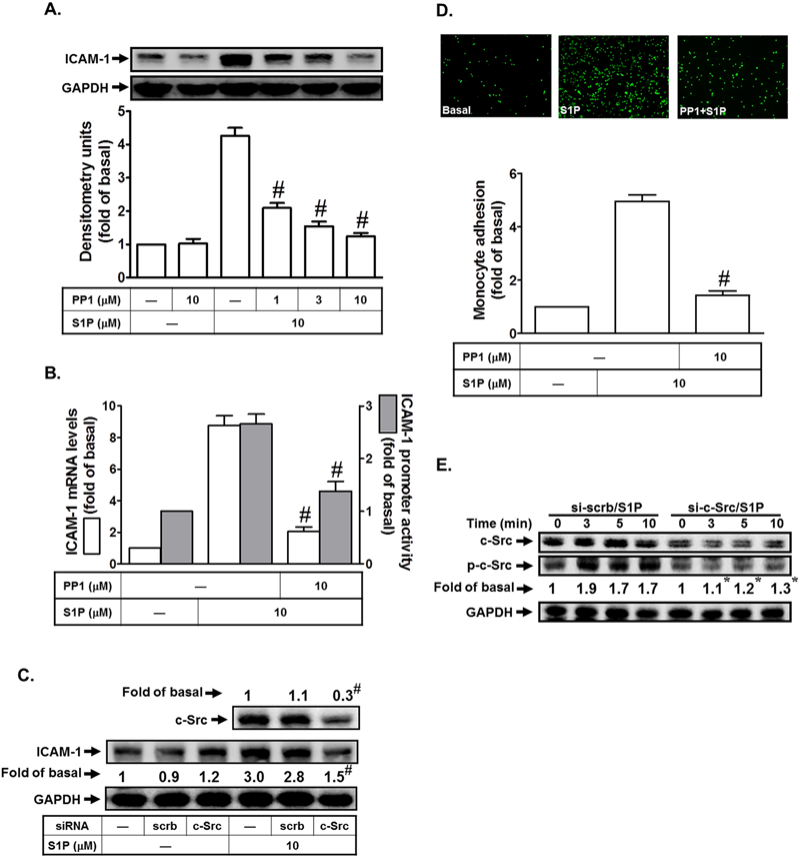
S1P induces ICAM-1 expression via c-Src. (A) HPAEpiCs were pretreated with PP1 for 1 h, and then incubated with S1P for 16 h. The ICAM-1 protein expression was determined by Western blot. (B) Cells were pretreated with PP1 (10 M) for 1 h, and then incubated with S1P for 4 h. The ICAM-1 mRNA expression and promoter activity were determined by real-time PCR and promoter assay, respectively. (C) Cells were transfected with siRNA of scrambled or c-Src, and then incubated with S1P (10 μM) for 16 h. The levels of c-Src and ICAM-1 proteins were determined by Western blot. (D) Cells were pretreated with PP1 (10 M) for 1 h, and then incubated with S1P for 16 h. The THP-1 cells adherence was measured. (E) Cells were transfected with siRNA of scrambled or c-Src, and then incubated with S1P for the indicated time intervals. The levels of phospho-c-Src protein were determined by Western blot. Data are expressed as mean (C, E) or mean±S.E.M (A, B, D) of three independent experiments. *P<0.05; ^#^ *P*<0.01, as compared with the cells exposed to S1P alone (A, B, D) or transfected with siRNA of scrambled+S1P (C, E).

c-Src, a common modulator participating in the crosstalk between the cytoplasmic protein tyrosine kinases and receptors, has been shown to mediate ICAM-1 expression in various cell types [9,10]. Thus, we investigated whether c-Src was involved in S1P-induced ICAM-1 expression in HPAEpiCs. As shown in Fig. 3A and B, pretreatment with the inhibitor of c-Src (PP1) markedly reduced S1P-induced ICAM-1 protein and mRNA expression and promoter activity. We confirmed the role of c-Src in S1P-induced ICAM-1 expression in HPAEpiCs by using c-Src siRNA. As shown in Fig. 3C, transfection with c-Src siRNA markedly reduced the c-Src protein expression, and then inhibited S1P-induced ICAM-1 expression. In addition, c-Src inhibition by PP1 also attenuated monocyte adhesion to HPAEpiCs challenged with S1P (Fig. 3D). Finally, we observed that S1P could enhance c-Src phosphorylation in a time-dependent manner in these cells, which was inhibited by c-Src siRNA (Fig. 3E). Taken together, we suggest that S1P induces ICAM-1 expression via a c-Src-dependent pathway in HPAEpiCs.

### S1P induces ICAM-1 expression via EGFR and PDGFR activation

**Fig 4:**
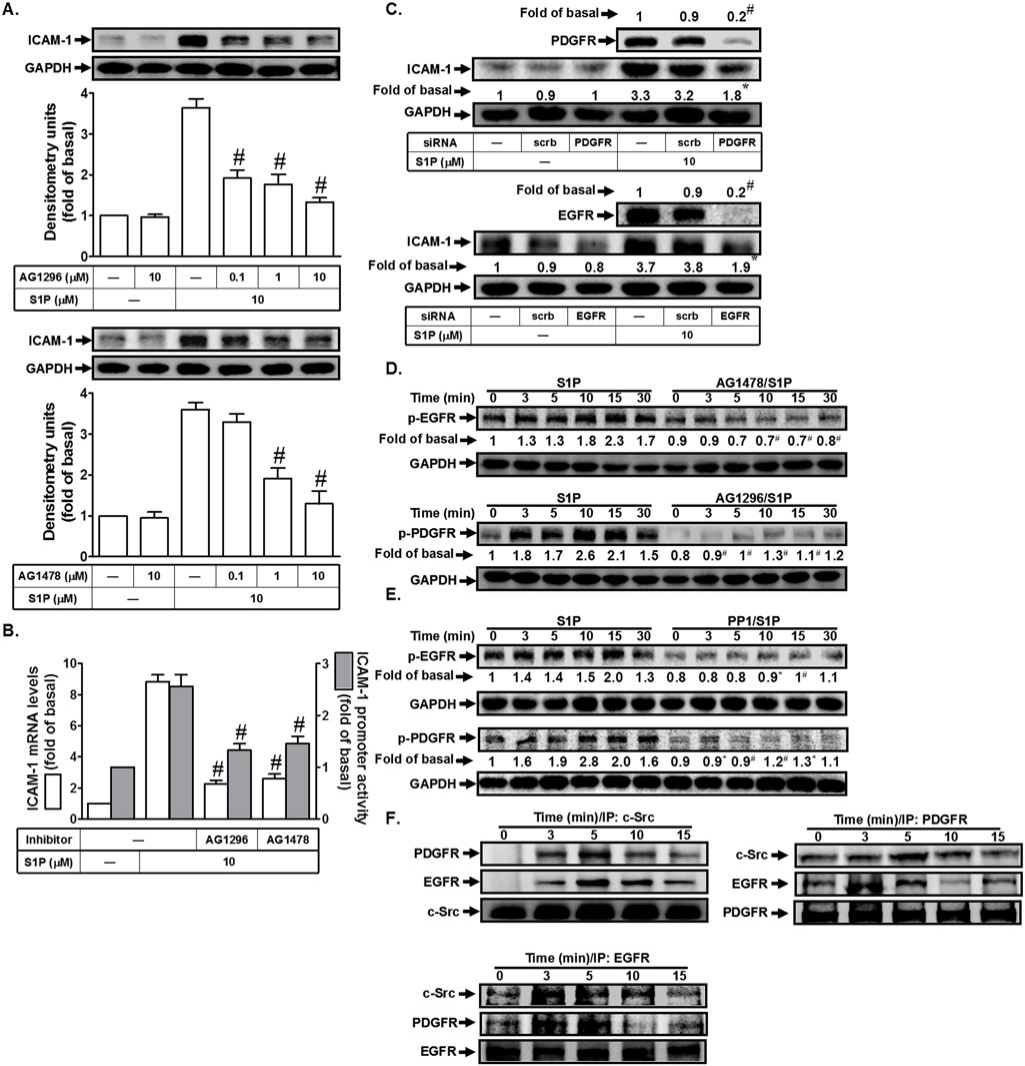
S1P induces ICAM-1 expression via EGFR and PDGFR. (A) HPAEpiCs were pretreated with AG1296 or AG1478 for 1 h, and then incubated with S1P for 16 h. The ICAM-1 protein expression was determined by Western blot. (B) Cells were pretreated with AG1296 (10 M) or AG1478 (10 μM) for 1 h, and then incubated with S1P for 4 h. The ICAM-1 mRNA expression and promoter activity were determined by real-time PCR and promoter assay, respectively. (C) Cells were transfected with siRNA of scrambled, EGFR, or PDGFR, and then incubated with S1P (10 μM) for 16 h. The levels of EGFR, PDGFR, and ICAM-1 proteins were determined by Western blot. (D, E) Cells were pretreated without or with AG1478, AG1296, or PP1 for 1 h, and then incubated with S1P for the indicated time intervals. The levels of phospho-EGFR or phospho-PDGFR were determined by Western blot. (F) Cells were treated with S1P for the indicated time intervals. The cell lysates were subjected to immunoprecipitation using an anti-c-Src, anti-EGFR, or anti-PDGFR antibody. The immunoprecipitates were analyzed by Western blot using an anti-PDGFR, anti-EGFR, or anti-c-Src antibody. Data are expressed as mean (C, D, E) or mean±S.E.M (A, B) of three independent experiments. *P<0.05; ^#^ *P*<0.01, as compared with the cells exposed to S1P alone (A, B, D, E) or transfected with siRNA of scrambled+S1P (C).

Previous studies indicated that c-Src regulates platelet-derived growth factor receptor (PDGFR) and epidermal growth factor receptor (EGFR) transactivation [11], which further promotes the expression of inflammatory genes. Thus, we investigated whether EGFR or PDGFR was involved in S1P-induced ICAM-1 expression in HPAEpiCs. As shown in Fig. 4A and B, pretreatment with the inhibitor of EGFR (AG1478) or PDGFR (AG1296) markedly reduced S1P-induced ICAM-1 protein expression, mRNA levels, and promoter activity. We confirmed the role of EGFR or PDGFR in S1P-induced ICAM-1 expression in HPAEpiCs by using siRNA of EGFR or PDGFR. As shown in Fig. 4C, transfection with EGFR or PDGFR siRNA markedly reduced the respective protein expression, and then inhibited S1P-induced ICAM-1 expression. We observed that S1P could enhance EGFR or PDGFR phosphorylation in a time-dependent manner in these cells, which was inhibited by AG1478 or AG1296, respectively. We further investigated the relationship of c-Src, EGFR, and PDGFR in S1P-stimulated HPAEpiCs. As shown in Fig. 4E, we found that pretreatment with PP1 markedly reduced S1P-stimulated EGFR and PDGFR phosphorylation, suggesting that c-Src plays as an upstream molecule in regulating S1P-stimulated EGFR and PDGFR phosphorylation. Finally, we investigated the physical association of EGFR, PDGFR, and c-Src in S1P-stimulated HPAEpiCs by immunoprecipitation using an anti-c-Src, anti-EGFR, or anti-PDGFR antibody and Western blot. As shown in Fig. 4F, we observed that S1P time-dependently induced the formation of a c-Src/EGFR/PDGFR complex in these cells. Taken together, we suggest that S1P-induced ICAM-1 expression is mediated through a c-Src/EGFR or a c-Src/PDGFR pathway in HPAEpiCs.

### S1P induces ICAM-1 expression via MAPKs

**Fig 5:**
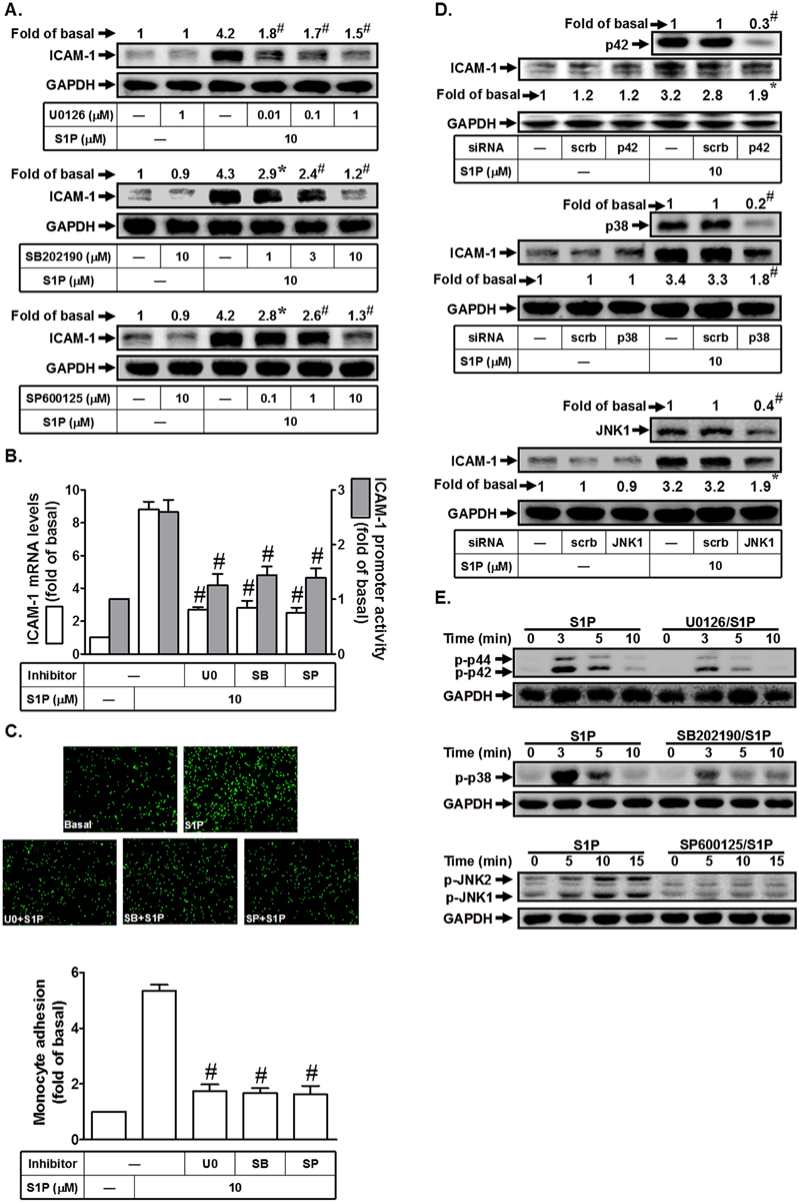
S1P induces ICAM-1 expression via MAPKs. (A) HPAEpiCs were pretreated with U0126, SB202190, or SP600125 for 1 h, and then incubated with S1P for 16 h. The ICAM-1 protein expression was determined by Western blot. (B) Cells were pretreated with SB202190 (10 M), U0126 (1 μM), or SP600125 (10 M) for 1 h, and then incubated with S1P for 4 h. The ICAM-1 mRNA expression and promoter activity were determined by real-time PCR and promoter assay, respectively. (C) Cells were pretreated with SB202190 (10 M), U0126 (1 μM), or SP600125 (10 M) for 1 h, and then incubated with S1P for 16 h. The THP-1 cells adherence was measured. (D) Cells were transfected with siRNA of scrambled, p38, p42, or JNK1, and then incubated with S1P (10 μM) for 16 h. The levels of p38, p42, JNK1, and ICAM-1 proteins were determined by Western blot. (E) Cells were pretreated without or with U0126 (1 μM), SB202190 (10 M), or SP600125 (10 M) for 1 h, and then incubated with S1P (10 M) for the indicated time intervals. The levels of phospho-p42/p44 MAPK, phospho-p38 MAPK, and phospho-JNK1/2 were determined by Western blot. Data are expressed as mean (A, D) or mean±S.E.M (B, C) of three independent experiments. *P<0.05; ^#^ *P*<0.01, as compared with the cells exposed to S1P alone (A, B, C) or transfected with siRNA of scrambled+S1P (D).

MAPK cascades are highly conserved signaling modules downstream of receptors/sensors that relay extracellular stimuli into intracellular responses in eukaryotes. MAPKs also have been shown to regulate S1P-induced inflammatory responses [12,13]. In this study, we found that pretreatment with the inhibitor of p38 MAPK (SB202190), MEK1/2 (U0126), or JNK1/2 (SP600125) significantly reduced S1P-induced ICAM-1 protein and mRNA expression and promoter activity (Fig. 5A and B). In addition, all these three inhibitors could attenuate monocyte adhesion to HPAEpiCs challenged with S1P (Fig. 5C). We further used siRNA of p38, p42, or JNK1 to confirm the roles of MAPKs in S1P-induced ICAM-1 expression in HPAEpiCs. As shown in Fig. 5D, transfection with siRNA of p38, p42, or JNK1 down-regulated the expression of respective proteins and markedly reduced S1P-induced ICAM-1 expression in HPAEpiCs. To investigate whether S1P-induced ICAM-1 expression was mediated through MAPKs activation, the phosphorylation of p42/p44 MAPK, JNK1/2, or p38 MAPK was observed in S1P-stimulated HPAEpiCs. As shown in Fig. 5E, S1P markedly stimulated the phosphorylation of these three MAPKs in a time-dependent manner, which was reduced by their respective inhibitors. Thus, we demonstrated that S1P-induced ICAM-1 expression was mediated through a MAPKs signaling pathway in HPAEpiCs.

### S1P induces c-Src/EGFR, PDGFR-dependent p42/p44 MAPK and p38 MAPK activation

**Fig 6:**
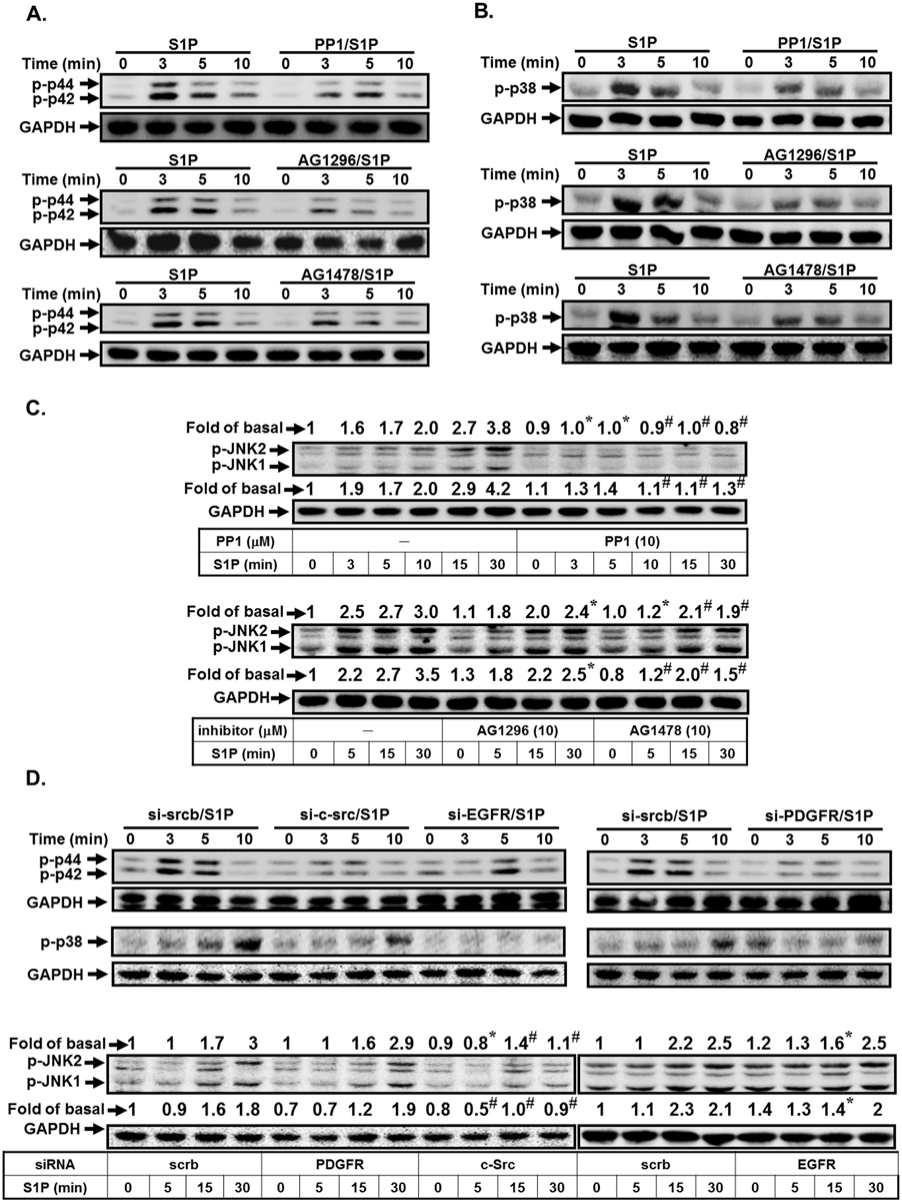
S1P induces c-Src-dependent p42/p44 MAPK and p38 MAPK activation. HPAEpiCs were pretreated without or with PP1, AG1478, or AG1296 for 1 h, and then incubated with S1P for the indicated time intervals. The levels of (A) phospho-p42/p44 MAPK, (B) phospho-p38 MAPK, or (C) phospho-JNK1/2 were determined by Western blot. Data are expressed as representatives of three independent experiments. (D) Cells were transfected with siRNA of scrambled, c-Src, EGFR, or PDGFR, and then incubated with S1P (10 μM) for the indicated time intervals. The levels of phospho-p38 MAPK, phospho-p42/p44 MAPK, phospho-JNK1/2, and GAPDH proteins were determined by Western blot.

Here, we investigated the relationship of c-Src, EGFR, PDGFR, and MAPKs in S1P-stimulated HPAEpiCs. As shown in Fig. 6A and B, S1P markedly induced p42/p44 MAPK and p38 MAPK phosphorylation in a time-dependent manner, which was inhibited by PP1, AG1478, or AG1296 in HPAEpiCs. However, we found that pretreatment with PP1 was attenuated S1P-induced JNK1/2 activation, but not AG1296 and AG1478 in these cells (Fig. 6C). To further confirm the roles of c-Src-dependent EGFR/PDGFR in S1P-induced MAPKs activation, siRNAs of c-Src, EGFR, and PDGFR were used. As shown in Fig. 6D, transfection with siRNA of c-Src was significantly reduced S1P-induced MAPKs activation. However, S1P-induced phosphorylation of p42/p44 MAPK and p38 MAPK but not JNK was significantly reduced by transfection with siRNA of EGFR, or PDGFR. Thus, we demonstrated that S1P stimulated p38 MAPK and p42/p44 MAPK phosphorylation via a c-Src/EGFR and PDGFR-dependent pathway.

S1P induces ICAM-1 expression via PI3K/Akt in HPAEpiCs

**Fig 7:**
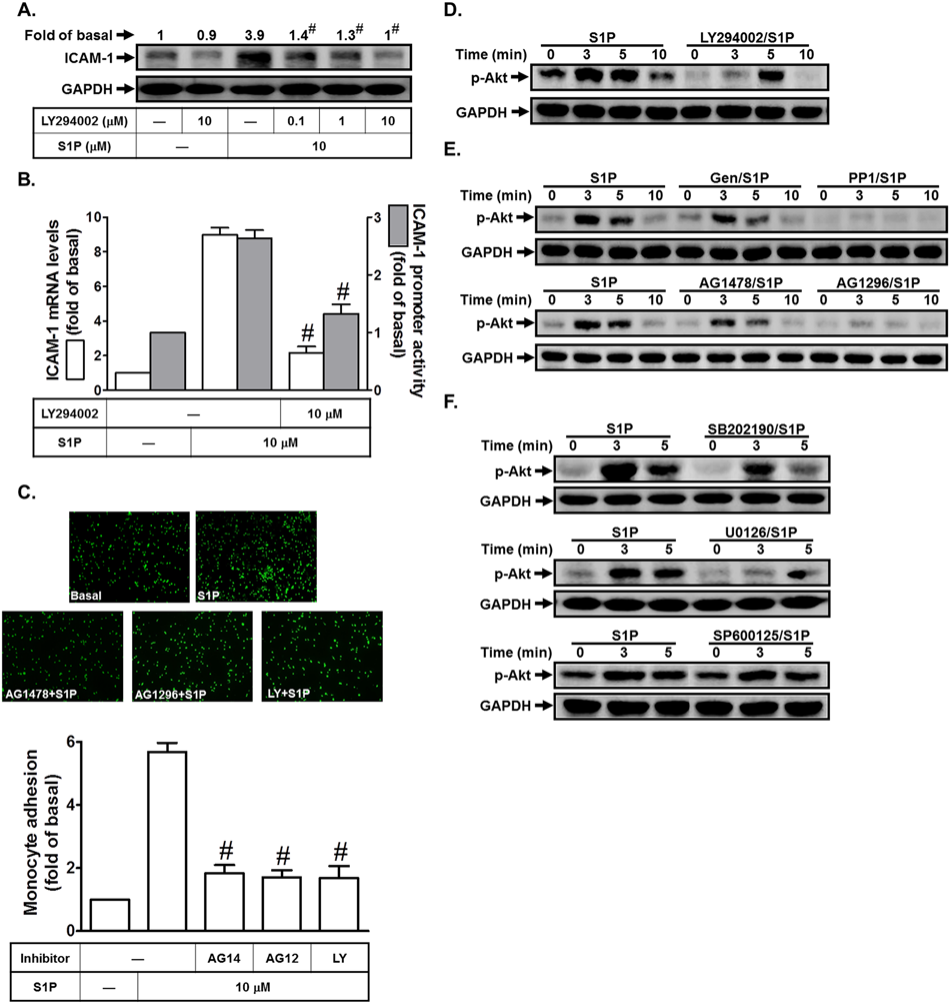
S1P induces ICAM-1 expression via PI3K/Akt. (A) HPAEpiCs were pretreated with LY294002 for 1 h, and then incubated with S1P for 16 h. The ICAM-1 protein expression was determined by Western blot. (B) Cells were pretreated with LY294002 (10 M) for 1 h, and then incubated with S1P for 4 h. The ICAM-1 mRNA expression and promoter activity were determined by real-time PCR and promoter assay, respectively. (C) Cells were pretreated with AG1478 (10 M), AG1296 (10 μM), or LY294002 (10 μM) for 1 h, and then incubated with S1P for 16 h. The THP-1 cells adherence was measured. (D-F) Cells were pretreated with LY294002, Genistein, PP1, AG1478, AG1296, SB202190, SP600125, or U0126 for 1 h, and then incubated with S1P for the indicated time intervals. The levels of phospho-Akt were determined by Western blot. Data are expressed as mean±S.E.M. of three independent experiments. ^#^ *P*<0.01, as compared with the cells exposed to S1P alone.

Previous studies indicated that PI3K/Akt regulates the expression of adhesion molecules in various cell types [10,14]. S1P has been shown to enhance Akt activation [15,16]. Thus, we investigated whether PI3K/Akt were involved in S1P-induced ICAM-1 expression in HPAEpiCs. In this study, we found that pretreatment with the inhibitor of PI3K (LY294002) significantly reduced S1P-enhanced ICAM-1 protein and mRNA expression and promoter activity (Fig. 7A and B). In addition, monocyte adhesion to HPAEpiCs challenged with S1P was also reduced by AG1478, AG1296, or LY294002 (Fig. 7C). We found that S1P markedly stimulated Akt phosphorylation in a time-dependent manner, which was inhibited by LY294002 (Fig. 7D). We further investigated the relationship of c-Src, EGFR, PDGFR, and Akt in S1P-stimulated HPAEpiCs. As shown in Fig. 7E, S1P-stimulated Akt phosphorylation was reduced by Genistein (an inhibitor of tyrosine protein kinases), PP1, AG1478, or AG1296 in these cells. On the other hand, we also observed the relationship between MAPKs and Akt in S1P-stimulated HPAEpiCs. As shown in Fig. 7F, we found that S1P-stimulated Akt phosphorylation was inhibited by SB202190 or U0126, but not SP600125. Indeed, we found that pretreatment with LY294002 had no effects on S1P-stimulated p42/p44 MAPK, JNK1/2, and p38 MAPK phosphorylation (data not shown). Thus, we suggested that S1P-stimulated Akt phosphorylation is mediated through p42/p44 MAPK and p38 MAPK in HPAEpiCs.

### S1P induces ICAM-1 expression via AP-1 in HPAEpiCs

**Fig 8:**
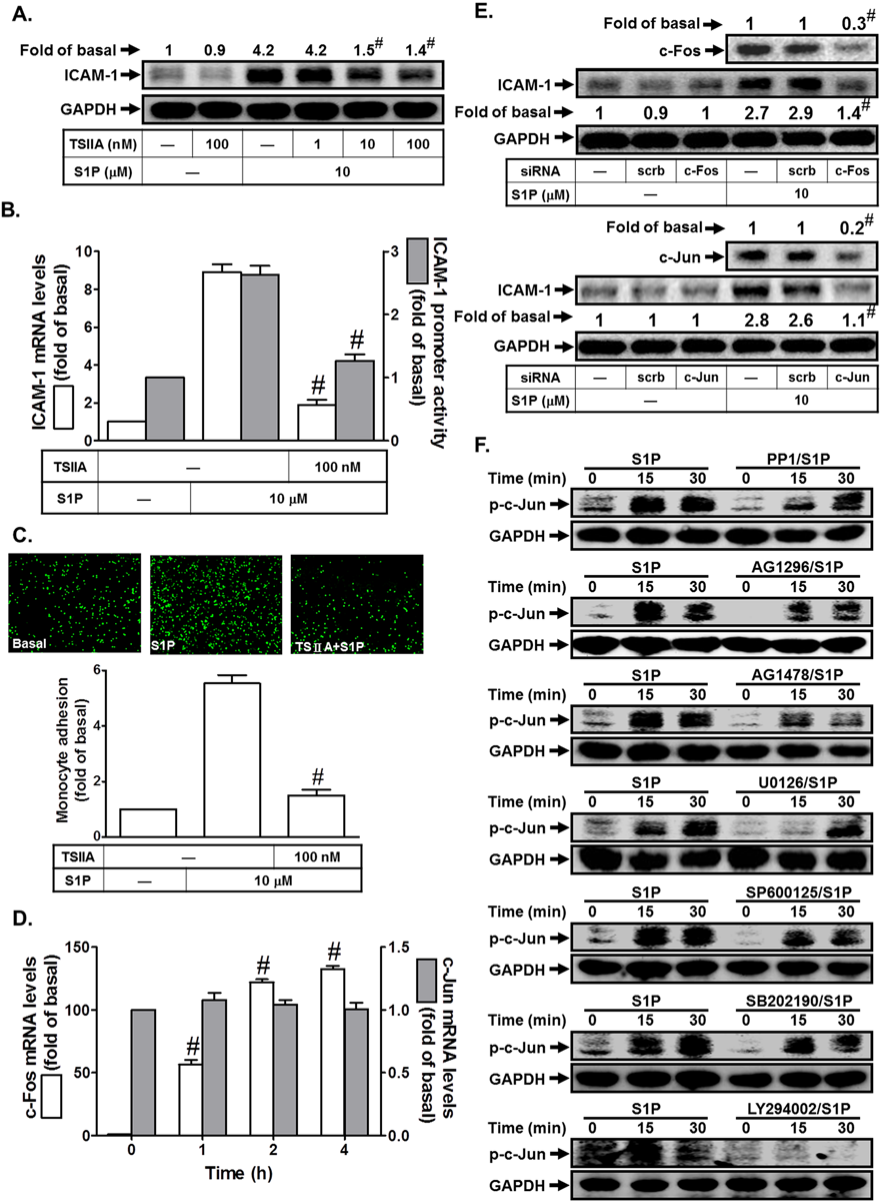
S1P induces ICAM-1 expression via AP-1. (A) HPAEpiCs were pretreated with Tanshinone IIA for 1 h, and then incubated with S1P for 16 h. The ICAM-1 protein expression was determined by Western blot. (B) Cells were pretreated with Tanshinone IIA (100 nM) for 1 h, and then incubated with S1P for 4 h. The ICAM-1 mRNA expression and promoter activity were determined by real-time PCR and promoter assay, respectively. (C) Cells were pretreated with Tanshinone IIA (100 nM) for 1 h, and then incubated with S1P for 16 h. The THP-1 cells adherence was measured. (D) Cells were treated with S1P for the indicated time intervals. The mRNA levels of c-Jun and c-Fos were determined by real-time PCR. (E) Cells were transfected with siRNA of scrambled, c-Jun, or c-Fos, and then incubated with S1P (10 μM) for 16 h. The levels of c-Fos, c-Jun, and ICAM-1 proteins were determined by Western blot. (F) Cells were pretreated without or with PP1, AG1296, AG1478, U0126, SP600125, SB202190, or U0126 for 1 h, and then incubated with S1P for the indicated time intervals. The levels of phospho-c-Jun were determined by Western blot. Data are expressed as mean (A, E) or mean±S.E.M. of three independent experiments. ^#^ *P*<0.01, as compared with the cells exposed to S1P alone (A-C), vehicle alone (D), or transfected with siRNA of scrambled+S1P (E).

The ICAM-1 promoter has been shown to contain several binding sequences for various transcription factors, including AP-1 [17]. To investigate whether AP-1 was involved in S1P-induced ICAM-1 expression, the inhibitor of AP-1 (Tanshinone IIA) was used. As shown in Fig. 8A and B, pretreatment with Tanshinone IIA markedly reduced S1P-induced ICAM-1 protein and mRNA expression and promoter activity. In addition, Tanshinone IIA also attenuated monocyte adhesion to HPAEpiCs challenged with S1P (Fig. 8C). AP-1 is a heterogeneous collection of dimeric transcription factors comprising Jun, Fos, and ATF subunits. Among AP-1 subunits, c-Jun and c-Fos are the most important transcriptional activators in inflammatory status. In this study, we found that S1P markedly induced c-Fos, but not c-Jun mRNA expression in these cells (Fig. 8D). To further confirm the roles of c-Jun and c-Fos in S1P-induced ICAM-1 expression in HPAEpiCs, as shown in Fig. 8E, transfection with c-Jun or c-Fos siRNA significantly reduced c-Jun or c-Fos protein expression, and then inhibited S1P-induced ICAM-1 expression in HPAEpiCs. Finally, we observed that S1P time-dependently stimulated c-Jun phosphorylation, which was reduced by PP1, AG1296, AG1478, U0126, SP600125, SB202190, or LY294002 (Fig. 8F). Taken together, we suggest that S1P induces ICAM-1 expression via an AP-1-dependent signaling in HPAEpiCs.

### S1P-evoked c-Src/EGFR and PDGFR/p38 MAPK and p42/p44 MAPK/Akt- or JNK1/2-dependent AP-1 activation are mediated via S1PR1/3

**Fig 9:**
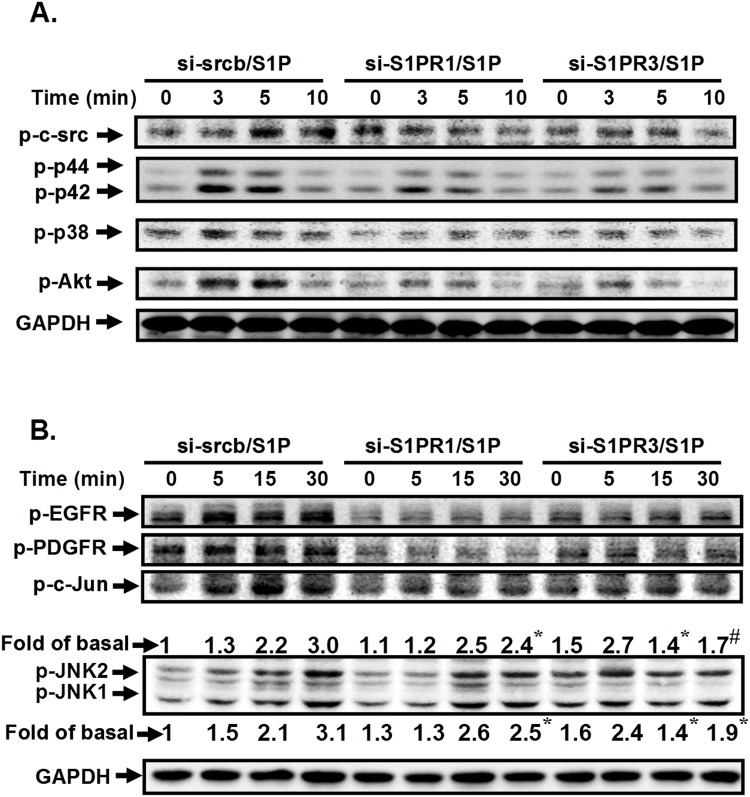
S1P stimulates c-Src/EGFR, PDGFR/p38 MAPK, p42/p44 MAPK/Akt- or JNK1/2-dependent AP-1 activation are mediated via S1PR1/3. (A) Cells were transfected with siRNA of scrambled, S1PR1, or S1PR3, and then incubated with S1P (10 μM) for the indicated time intervals. The levels of phospho-c-Src, phospho-EGFR, phospho-PDGFR, phospho-p38 MAPK, phospho-p42/p44 MAPK, phospho-JNK1/2, phospho-c-Jun, and GAPDH proteins were determined by Western blot. The figure represents one of three individual experiments (n = 3).

S1P-induced ICAM-1 expression was mediated through c-Src/EGFR/PDGFR/p38 MAPK, p42/p44 MAPK/Akt- or JNK1/2-dependent AP-1 activation. However, whether these signaling pathways regulated by distinct S1PRs are still unclear. As shown in Fig. 9, S1P-stimulated phosphorylation of c-Src, EGFR, PDGFR, Akt, MAPKs, and AP-1 was reduced by transfection with siRNA of S1PR1 or S1PR3, suggesting that S1P-induced ICAM-1 expression is mediated through S1PR1/3/c-Src/EGFR, PDGFR/p38 MAPK, p42/p44 MAPK/Akt-dependent AP-1 activation in HPAEpiCs.

## Discussion

Asthma and COPD are respiratory disorders characterized by various degrees of inflammation and tissue remodeling. S1P is a bioactive sphingolipid metabolite that plays important roles in allergic responses, including asthma and anaphylaxis [4]. Moreover, S1P has been shown to play a key role in inflammation via adhesion molecules, such as ICAM-1 induction, and then causes lung injury. However, the molecular mechanisms by which S1P induces ICAM-1-dependent monocyte adhesion are not fully understood in HPAEpiCs. The present study clearly demonstrated that S1P-induced ICAM-1 expression was regulated via S1PR1/3/c-Src/EGFR, PDGFR/p38 MAPK, p42/p44 MAPK/Akt-dependent AP-1 activation. Genetic silencing through transfection with siRNA of S1PR1, S1PR3, c-Src, EGFR, PDGFR, p38, p42, JNK1, c-Jun, or c-Fos and pretreatment with the inhibitor of S1PR1 (W123), S1PR3 (CAY10444), c-Src (PP1), EGFR (AG1478), PDGFR (AG1296), MEK1/2 (U0126), p38 MAPK (SB202190), JNK1/2 (SP600125), PI3K (LY294002), or AP-1 (Tanshinone IIA) attenuated S1P-induced ICAM-1 expression and monocyte adhesion. Therefore, activation of S1P receptors by S1P causes inflammatory responses through ICAM-1 up-regulation.

Several lines of evidence have reported that S1P-induced diverse biological effects are mediated through S1PRs [20]. Moreover, S1PR1, S1PR2, and S1PR3 have been shown to be expressed on various cell types [21,22]. Indeed, we also found that S1PR1, S1PR2, and S1PR3 are expressed on HPAEpiCs. Zhang et al. indicated that S1PR2-mediated NF-κB activation contributes to TNF-α-induced VCAM-1 and ICAM-1 expression in endothelial cells [23]. Interestingly, in our study, we demonstrated that the inhibition of S1PR1 or S1PR3, but not S1PR2, significantly attenuated S1P-induced ICAM-1 expression in HPAEpiCs. Thus, we suggest that S1PR1 and S1PR3 mainly play key roles in S1P-induced ICAM-1 expression in these cells. The non-receptor tyrosine kinases of the Src family (SFK) play important roles in signal transduction induced by a large variety of extracellular stimuli [18]. SFKs are signaling enzymes that have long been recognized to regulate critical cellular processes, such as proliferation, survival, migration, and metastasis [1]. Moreover, Chang et al. showed that IFN-γ induces ICAM-1 expression via PKC α/c-Src activation in human NCI-H292 epithelial cells [24]. In addition, we previously indicated that TNF-α induces ICAM-1 expression via a c-Src signaling in human airway smooth muscle cells [9]. Here, we observed that S1P-induced ICAM-1 expression and monocyte adhesion was attenuated by a c-Src inhibitor PP1. Several studies have reported that c-Src is an essential component for cytokine-stimulated PDGFR or EGFR transactivation via the phosphorylation of cytoplasmic domains of EGFR or PDGFR [11,18]. The PDGF family of growth factors consists of five different disulphide-linked dimers built up of four different polypeptide chains encoded by four different genes. These isoforms, PDGF-AA, PDGF-AB, PDGF-BB, PDGF-CC, and PDGF-DD, act via two receptor tyrosine kinases, PDGF receptors α and β [18]. The classic PDGFs, PDGF-A and PDGF-B, undergo intracellular activation during transport in the exocytic pathway for subsequent secretion, while the novel PDGFs, PDGF-C and PDGF-D, are secreted as latent factors that require activation by extracellular proteases [18]. EGFR exists on the cell surface and is activated by binding of its specific ligands, including EGF and transforming growth factor- α (TGF- α). Moreover, activation of EGFR and PDGFR has been shown to induce respiratory system inflammation [1,2]. Here, we established that S1P induced ICAM-1-dependent monocyte adhesion via c-Src/EGFR and c-Src/PDGFR activation in HPAEpiCs. In addition, we also demonstrated that S1P could induce the formation of a c-Src/EGFR/PDGFR complex in these cells. Although the detailed protein-protein interactions among c-Src, EGFR, and PDGFR are not known, our results are the first time to show a novel role of c-Src/EGFR/PDGFR complex formation in S1P-induced ICAM-1 expression in HPAEpiCs. In the future, we will further determine which domains of c-Src, EGFR, and PDGFR are involved in protein-protein interactions caused by S1P.

The MAPKs regulate diverse cellular programs by relaying extracellular signals to intracellular responses. In mammals, there are more than a dozen MAPK enzymes that coordinately regulate cell proliferation, differentiation, motility, and survival. The best known are the conventional MAPKs, including p42/p44 MAPK, JNK1/2, and p38 MAPK [2]. MAPKs also have been shown to regulate S1P-induced inflammatory responses [12,13]. In addition, MAPKs also have been shown to regulate ICAM-1 induction and monocyte adhesion in response to various stimuli [3,17]. Indeed, in HPAEpiCs, we found that all these three MAPKs were involved in ICAM-1 expression and monocyte adhesion induced by S1P. Thus, we suggest that MAPKs play key roles in S1P-induced inflammatory responses. Lin et al. indicated that thrombin-induced NF-κB activation is mediated by a c-Src-dependent p42/p44 MAPK pathway in lung epithelial cells [25]. Our group also showed that c-Src-dependent MAPKs/AP-1 activation is involved in TNF-α-induced matrix metalloproteinase-9 expression in rat heart-derived H9c2 cells [18]. In this study, we observed that the inhibition of c-Src markedly reduced S1P-induced p42/p44 MAPK, p38 MAPK, and JNK1/2, activation in HPAEpiCs. On the other hand, we also showed that S1P induced p42/p44 MAPK and p38 MAPK, but not JNK1/2, phosphorylation via c-Src/EGFR- and PDGFR-dependent cascade.

The PI3Ks are a conserved family of signal transduction enzymes that are involved in cellular activation, inflammatory responses, chemotaxis, and apoptosis. PI3K/Akt have been shown to be a downstream component of EGFR or PDGFR activated by different stimuli in various cell types [11,18]. This is confirmed by our observation that S1P-induced Akt phosphorylation was reduced through the inhibition of c-Src, EGFR, or PDGFR. On the other hand, we found that pretreatment with LY294002 inhibited S1P-induced ICAM-1 expression, consistent with the results obtained with that ICAM-1 expression was mediated via a PI3K/Akt cascade in IL-1β-challenged A549 cells [10]. PI3K/Akt has been shown to regulate MAPKs activation in response to various stimuli, such as Japanese encephalitis virus and TNF-α [18,26]. In this study, we found that S1P-induced MAPKs activation was not regulated via PI3K/Akt (data not shown). Interestingly, we observed that S1P-induced Akt phosphorylation was reduced by SB202190 or U0126, but not SP600125, suggesting that S1P induced p38 MAPK- or p42/p44 MAPK-dependent Akt activation in HPAEpiCs. Indeed, Takahashi et al. showed that VEGF may stimulate PI3K/Akt through activation of the PKC and p42/p44 MAPK pathway in hepatic stellate cells [27]. Shi et al. also indicated that inhibition of p38 MAPK decreases Akt phosphorylation in proteasome inhibitors-stimulated breast carcinoma cells [28]. Thus, we suggest that in HPAEpiCs, S1P-stimulated p38 MAPK and p42/p44 MAPK phosphorylation plays key roles in mediating Akt activation leading to ICAM-1 expression.

It has been well established that inflammatory responses following exposure to extracellular stimuli are highly dependent on activation of AP-1, which plays an important role in the expression of several target genes. The ICAM-1 promoter has been shown to contain several binding sequences for various transcription factors, including AP-1 [17]. These studies suggest that AP-1 plays a critical role in the regulation of ICAM-1 expression in the inflammatory responses. Moreover, we found that AP-1 inhibition could reduce S1P-induced ICAM-1 expression. In S1P-stimulated HPAEpiCs, c-Fos mRNA expression was up-regulated. In addition, S1P also stimulated c-Jun phosphorylation in these cells. However, S1P had no effects on c-Jun mRNA levels. Previous studies indicated that AP-1 is regulated by various signaling components, such as c-Src and MAPKs [17,18,26], consistent with our results indicating that in HPAEpiCs, S1P stimulated c-Jun phosphorylation via a c-Src/EGFR and PDGFR/p42/p44 MAPK and p38 MAPK/PI3K/Akt- or JNK1/2-dependent pathway.

**Fig 10:**
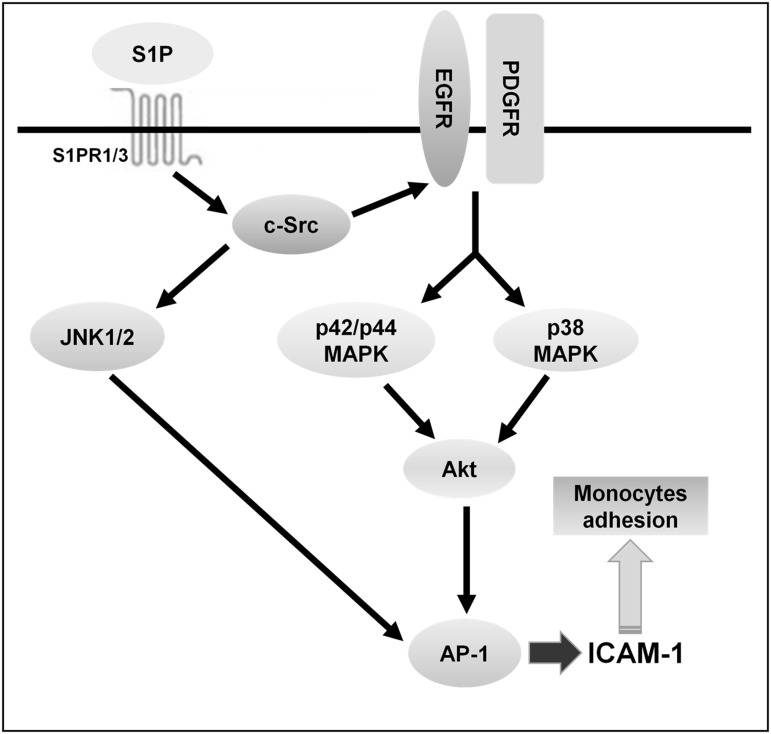
Proposed model to illustrate the signaling pathways involved in ICAM-1 expression and monocyte adhesion in HPAEpiCs challenged with S1P. S1P-induced ICAM-1 expression and monocyte adhesion were mediated through S1PR1/3/c-Src/EGFR and PDGFR/p38 MAPK and p42/p44 MAPK/Akt- or S1PR1/3/JNK1/2-dependent AP-1 activation.

In summary, as depicted in Fig. 10, our results showed that in HPAEpiCs, S1P-induced ICAM-1 expression and monocyte adhesion were mediated through S1PR1/3/c-Src/EGFR and PDGFR/p38 MAPK and p42/p44 MAPK/Akt- or S1PR1/3/c-Src/JNK1/2-dependent AP-1 activation. These results provide new insights into the mechanisms of S1P-induced the expression of ICAM-1 and monocyte adhesion and thus exaggerate the inflammatory responses. Increased understanding of signaling mechanisms underlying ICAM-1 gene regulation will create opportunities for the development of anti-inflammation therapeutic strategies.
